# Effects of *Ginkgo biloba* on chemically-induced mammary tumors in rats receiving tamoxifen

**DOI:** 10.1186/1472-6882-13-93

**Published:** 2013-05-01

**Authors:** Marcos Correa Dias, Kelly Silva Furtado, Maria Aparecida Marchesan Rodrigues, Luís Fernando Barbisan

**Affiliations:** 1 Post-Graduation Program in Pathology, School of Medicine, UNESP - Univ Estadual Paulista, Botucatu, SP 18618-970, Brazil; 2 Department of Pathology, School of Medicine, UNESP - Univ Estadual Paulista, Botucatu, SP, 18618-970, Brazil; 3 Department of Morphology, UNESP - Univ Estadual Paulista, Institute of Biosciences, Botucatu, SP, 18618-970, Brazil

**Keywords:** Tamoxifen, Selective estrogen receptor modulator, Complementary and alternative medicine, Rat, Mammary carcinogenesis

## Abstract

**Background:**

*Ginkgo biloba* extract (GbE) is used extensively by breast cancer patients undergoing treatment with Tamoxifen (TAM). Thus, the present study investigated the effects of GbE in female Sprague–Dawley (SD) rats bearing chemically-induced mammary tumors and receiving TAM.

**Methods:**

Animals bearing mammary tumors (≥1 cm in diameter) were divided into four groups: TAM [10 mg/kg, intragastrically (i.g.)], TAM plus GbE [50 and 100 mg/kg, intraperitoneally (i.p.)] or an untreated control group. After 4 weeks, the therapeutic efficacy of the different treatments was evaluated by measuring the tumor volume (cm^3^) and the proportions of each tumor that were alive, necrotic or degenerative (mm^2^). In addition, labeling indexes (LI%) were calculated for cell proliferation (PCNA LI%) and apoptosis (cleaved caspase-3 LI%), expression of estrogen receptor-alpha (ER-α) and p63 biomarkers.

**Results:**

Overall, the tumor volume and the PCNA LI% within live tumor areas were reduced by 83% and 99%, respectively, in all TAM-treated groups when compared to the untreated control group. GbE treatment (100 mg/kg) reduced the proportions of live (24.8%) and necrotic areas (2.9%) (p = 0.046 and p = 0.038, respectively) and significantly increased the proportion of degenerative areas (72.9%) (p = 0.004) in mammary tumors when compared to the group treated only with TAM. The expression of ER-α, p63 and cleaved caspase-3 in live tumor tissues was not modified by GbE treatment.

**Conclusions:**

Co-treatment with 100 mg/kg GbE presented a slightly beneficial effect on the therapeutic efficacy of TAM in female SD rats bearing mammary tumors.

## Background

The majority of breast cancers are estrogen-dependent disease with increasing morbidity and mortality rates in most western societies over the last few decades [1-3]. The most common therapeutic strategies for breast cancer, including excision surgery (mastectomy), radiotherapy, chemotherapy, monoclonal antibodies and endocrine therapies, impact on women’s quality of life [4]. As an endocrine adjuvant therapy, anti-estrogenic drugs target the estrogen receptor (ER)-dependent intracellular response by directly binding to and inhibiting ERs (Selective Estrogen Receptor Modulators, SERMs) or by down-regulating the synthesis of endogenous estrogens (Aromatase Inhibitors, AIs) [5,6]. The SERMs, which include TAM and Raloxifen, have been established as gold standard first-line therapies for estrogen-dependent breast cancers [7]. TAM has been found to reduce the incidence of breast cancer in high-risk pre- and post-menopausal women and to enhance disease-free survival and reduce disease recurrence [8]. However, an extensive evaluation of TAM use has revealed some side effects such as increased risk for endometrial cancer, deep-vein thrombosis and pulmonary embolism [9-11]. These important side effects have resulted in the use of alternative treatments such as complementary and alternative medicine (CAM).

Popular interest in CAM has grown rapidly over the past decade in the western world [12]. Commercial advertisements, many of which promise cures, have stimulated the consumption of CAM treatments including herbal, vitamin and nutritional supplements. The use of CAM is more common among cancer patients than among the general population [12]. Recent reports estimate that 7-64% of chemotherapy patients in 26 cohort studies worldwide have used herbal supplements [13,14], and up to 72% of these patients did not inform their physician about their concomitant CAM usage [15]. Although the use of herbal or ‘natural’ drugs is rapidly growing, most of these promising therapies remain poorly understood, and limited scientific evidence regarding their efficacy and safety is available [16]. Thus, preclinical and clinical studies are necessary to evaluate the safety and efficacy of each CAM alone and/or in combination with prescription drug therapies.

*Ginkgo biloba* extract (GbE) is a well-established medicinal herb extensively used as a CAM in diseases including breast cancer [17]. GbE is a complex mixture of over 300 compounds primarily composed of flavonoid glycosides and terpenoids such as ginkgolides and bilobalides [17,18]. GbE has been used for the prevention and treatment of brain disorders, systemic circulatory disorders, memory loss and Alzheimer’s disease [17,19,20]. In fact, many molecules within GbE have been shown to exhibit pharmacological properties such as cell cycle regulatory, antioxidant, anti-proliferative, anti-angiogenic and anti-estrogenic activities [21]. As GbE is used extensively as a CAM [17] and is used by breast cancer patients undergoing treatment with TAM [22], the present study was designed to investigate the effects of *Ginkgo biloba* extract in a chemically induced mammary tumor model in female SD rats treated with Tamoxifen.

## Methods

### Animals and treatments

The animals used in this study were handled in accordance with the ethical principles for animal research adopted by the Brazilian College of Animal Experimentation (COBEA). The protocols used here were approved by the Botucatu School of Medicine Ethical Committee for Animal Research (protocol no. 51/08- Commission of Ethics in Animal Experimentation, CEEA). Four-week-old female Sprague–Dawley (SD) rats were purchased from CEMIB-UNICAMP (Campinas- SP, Brazil). All of the animals were housed in polypropylene cages (four animals/cage) covered with metallic grids in a room maintained at 22 ± 2°C and 55 ± 10% humidity with a 12-hr light–dark cycle. Food and water consumption were measured twice a week, and the animals were weighed once a week during the entire 4-week treatment period.

Tamoxifen citrate (TAM, Nolvadex^®^) was purchased from AstraZeneca UK Limited (Macclesfield, Cheshire, UK). The covered tablets were grasped in a melting pot, diluted in canola oil (3 mg/ml) and then orally administered at dose of 10 mg/kg [23]. *Ginkgo biloba* leaf extract (GbE, code 500821) was purchased from CentroFlora Group (Botucatu-SP-Brazil). GbE was obtained by hydroalcoholic extraction using a spray dryer system and contained 24% flavone glycosides (i.e., quercetin, kaempferol and isorhamnetin) and 6% terpene trilactones (i.e., bilobalide and ginkgolide A, B and C) as evaluated by High Performance Liquid Chromatography (HPLC) [24]. GbE was pre-diluted in a 10% ethanol-water solution and heated for 3 min at 40°C to evaporate the ethanol. GbE was administered intraperitoneally at doses of 50 and 100 mg/kg, which correspond to approximately 10x the therapeutic dose in humans [25].

### Experimental procedures and tissue processing

At 51 days of age, female SD rats were given a single dose of 7,12-dimethyl-benz(a)anthracene (DMBA, 80 mg/kg, i.g.) [24]. Female SD rats bearing palpable mammary tumors (≥1 cm in diameter) were randomly allocated into four groups (10 rats/group): TAM-treated (10 mg/kg, i.g.), TAM plus GbE-treated (50 mg/kg, i.p.), TAM plus GbE-treated (100 mg/kg, i.p.) and TAM vehicle plus GbE vehicle-treated (canola oil and water, respectively).

Immediately before the beginning of treatments, all animals bearing palpable tumors were submitted to excision biopsies under sodium pentobarbital anesthesia (30 mg/kg, i.p.). The excision biopsies were performed to evaluate the histopathological pattern of the tumor cell proliferation and apoptosis indexes and the expression of estrogen receptor α (ER-α) and p63. After 4 weeks of treatment, the animals were euthanized with CO_2_, and blood samples were collected for the analysis of alanine aminotransferase (ALT, U/l) and estradiol (E2, pg/ml) using Ortho-Clinical Diagnostics Reagents (Johnson & Johnson Co., SP, Brazil). The liver, kidneys, ovaries and tumor samples were collected during necropsy, fixed in 4% formalin, embedded in paraffin, sectioned at 5 μm and stained with hematoxylin-eosin (HE) for histopathological analysis. The biopsies (collected at the beginning of the experiment) and tumor samples (collected at necropsy) were immunohistochemically stained for proliferating cell nuclear antigen (PCNA), cleaved caspase-3, ER-α and p63.

### Measurements of mammary tumor volume and area

Mammary tumors were measured macroscopically in three dimensions using a caliper rule and their volume (cm^3^) was calculated according to the ellipsis volume formula: 3/4π *×* width *×* thickness *×* depth [26]. All tumor measures were obtained under sodium pentobarbital anesthesia at the beginning of the experiment, immediately before the excision biopsy, and after the 4-week treatment period. The rates of tumor growth were determined by calculating the difference between the final and initial volumes.

Tumor areas were morphometrically assessed in the HE staining slides and the sizes of live, degenerative and necrotic areas were measured within the total tumor area [27]. This analysis was performed at *200x* magnification using a Nikon photomicroscope (Microphot-FXA) connected to a KS-300 apparatus (Kontron Elektronic, Germany). The percentual area fraction of live, degenerative and necrotic areas in the representative sections cut through the middle of the tumor were estimated by dividing the size of each tissue area by the total tumor section volume [27]. The total mammary tumor section areas were measured using a special Macro-Stand device (support with Canon TV zoom lens V6×16/16-100 mm plus a Canon 58 mm close-up 240 lens connected to a CCD black-and-white video camera module with a Sony DC-777 camera unit) connected to a KS-300.

### Immunohistochemical procedure for PCNA, caspase-3, ER-α and p63

The deparaffinized 5 μm serial mammary tumor sections on poly-L-lysine-coated slides were first subjected to antigen retrieval by heating the slides in 0.01 M citrate buffer (pH 6.0) in a pressure cooker (Pascal, DakoCytomation, USA) or a microwave (3 × 5 min). Endogenous peroxidase was blocked with 3% H_2_O_2_ in phosphate-buffered saline (PBS) and nonspecific binding was blocked with 3% nonfat milk. The slides were then incubated overnight at 4°C with the following primary antibodies: mouse monoclonal anti-ER-α (clone 6F11, BioCare Medical–Concord, CA, USA, 1:50 dilution), mouse monoclonal anti-p63 (clone 4A4, DakoCytomation, Denmark A/S, Glostruo, Denmark, 1:75 dilution), mouse monoclonal anti-PCNA (clone PC10, DakoCytomation Denmark A/S, Glostrup, Denmark, 1:200 dilution) or rabbit polyclonal anti-cleaved caspase-3 (clone Asp 175 rabbit, Cell Signaling Technology, Inc., Danvers, MA, USA 1:100 dilution). The slides were then incubated with a biotinylated secondary anti-mouse antibody (Vector Laboratories, Inc., Burlingame, CA, USA, 1:200 dilution) and streptavidin-biotin-peroxidase solution (TissuGnost Kit, Merck, Darmstadt, Germany, 1:1:50 dilution). Chromogen color development was accomplished by 3,3-diaminobenzidine-tetrahydrochroride (DAB, Sigma-Aldrich Co., St. Louis MO, USA) local precipitation at the sites of peroxidase binding to the mammary tumor samples. Finally, the slides were counterstained with Harris’ hematoxylin, dehydrated and analyzed by optical microscopy. Negative controls for all of the immunoreactions were processed in adjacent sections by omitting the incubation with the primary antibodies.

The cell proliferation (PCNA LI%) and apoptosis (cleaved caspase-3 LI%) indexes and the expression of estrogen receptor-α (ER-α) and p63 (myoepithelial cells) were determined by calculating the percentage of PCNA^+^, cleaved caspase-3^+^, ER-α^+^ and p63^+^ cells among the total number of in 10–20 random microscopic fields analyzed in each tumor (~135 cells/field). All of these immunohistochemical analyses were performed in the live tumor tissues.

### Statistical analysis

The statistical analysis was performed using Jandel Sigma Stat software (Jandel Corporation, San Rafael, CA, USA). The data were analyzed by ANOVA when the results showed a normal distribution or by the Kruskal-Wallis test when they did not. Differences among the groups were analyzed by the Tukey or Student-Newman-Keuls methods. Biopsies were compared with tumors by performing t-tests for dependent variables. Differences were defined as statistically significant when p < 0.05.

## Results

### General observations

Food and water consumption (data not shown), body weight gain, relative liver, kidney and ovarian weights and serum levels of ALT and E2 did not differ among the different groups after 4 weeks of treatment with TAM and/or GbE (Table 1). The liver, kidney and ovaries did not present significant histopathological alterations associated with the different treatments (data not shown).

**Table 1:** **General parameters in the experimental groups after 4 weeks of treatment**^**1**^

	**Experimental groups**^**3**^			
**Parameters**^**2**^	**(G1)TAM**	**(G2) TAM + GbE50**	**(G3) TAM + GbE100**	**(G4) Untreated control**
*General parameters*				
Animal number (n)	10/9^4^	10/7	10/7	10/5
Tumor number (n)	11^5^	9	9	7
Initial body weight (g)	267.67 ± 3.25	257.25 ± 4.89	261.80 ± 7.89	283.0 ± 2.83
Final body weight (g)	291.33 ± 4.18	287.17 ± 7.99	287.17 ± 4.40	293.40 ± 3.71
Body weight gain (g)	23.43 ± 5.46	31.17 ± 4.71	28.80 ± 9.75	20.0 ± 7.93
Liver relative weight (g)	5.13 ± 0.57	4.38 ± 0.46	4.15 ± 0.67	4.53 ± 0.24
Left ovarian relative weight (mg)	20.0 ± 6.0	17.0 ± 2.64	13.01 ± 2.64	19.0 ± 3.12
Right ovarian relative weight (mg)	19.0 ± 5.0	13.0 ± 3.01	19.01 ± 5.56	17.0 ± 1.78
Initial tumor volume (cm^3^)	2.23 ± 0.49	2.64 ± 0.93	1.95 ± 0.46	2.09 ± 0.82
Final tumor volume (cm^3^)	0.85 ± 0.41*	0.83 ± 0.47*	0.57 ± 0.12*	4.73 ± 1.45
*Biochemical dosage*				
ALT (u/l)	37.17 ± 3.59	47.83 ± 4.24	47.0 ± 5.47	35.75 ± 7.68
E2 (ρg/ml)	57.80 ± 4.42	42.83 ± 3.24	34.0 ± 6.34	38.0 ± 6.65

^1^Data are expressed as mean ± SEM; ^2^*ALT*, Alanine amino transferase; *E2*, Estradiol; ^3^*TAM*, Tamoxifen (10 mg/Kg, i.p.); *GbE50*-*100*, *Ginkgo biloba *extract (50 or 100 mg/Kg, i.p.); ^4 ^The difference between initial and final number of rats/group was due to the dead or sacrifice of moribund animals (05 animals in untreated group) or development of mammary tumor with less than 5% of positivity for ER-α (01 and 03 animals in TAM alone and TAM + GbE50 respectively) or complete regression of tumors (03 animals in TAM + GbE100). ^5 ^Two animals from each group presented two mammary tumors before the beginning of treatments.

*Statistical difference when compared to the untreated group, p < 0.001.

### Mammary tumor volume and morphometric analyzes

**Figure 1:**
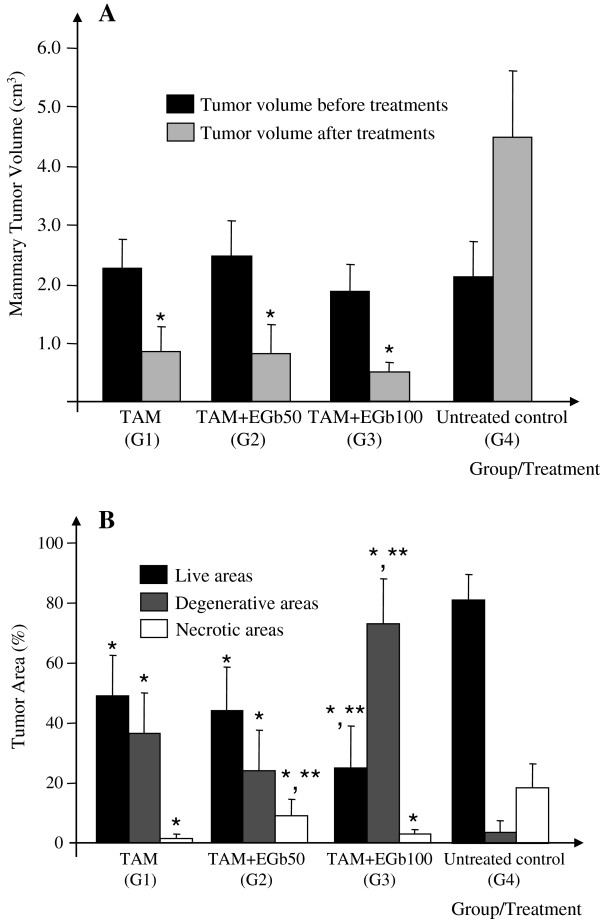
**Analysis of mammary tumor volume (before and after treatments) and tumor live, degenerative or necrotic areas (after treatments): (A) Mammary tumor volume at beginning of treatments (referential biopsies) and following 4 weeks of treatment with tamoxifen and *Gingko biloba *extract (tumors). **TAM = tamoxifen (10 mg/kg, i.p.); GbE 50-100 = *Ginkgo biloba *extract (50 or 100 mg/kg, i.g.). (**B**) Results of morphometric analysis of the proportion of the tumor tissue defined as live (%), degenerative (%) and necrotic (%) areas in all experimental groups after 4 weeks of treatment with tamoxifen and *Gingko biloba *extract. *,** Different from untreated control group and TAM alone group, 0.01 < p < 0.05), respectively.

Figure 1A shows the mammary tumor volume before and after 4 weeks of treatment. All TAM-treated groups presented a significant reduction in mammary tumor volume at the end of the treatment period compared to the untreated control group (p < 0.001) (Table 1, Figure 1A). The mean volume of the tumors in all of the TAM-treated groups was reduced by approximately 83% after treatment, whereas the mean volume of the tumors in the untreated control group increased by 178.7% during the treatment period. Co-treatment with either dose of GbE (50 or 100 mg/kg) did not significantly affect the mammary tumor regression induced by short-term TAM treatment (Table 1, Figure 1A). Also, some non-palpable tumors were detected at necropsy, as follows: 02 tumors in the TAM alone group and 02 tumors in TAM + GbE50 group.

**Figure 2:**
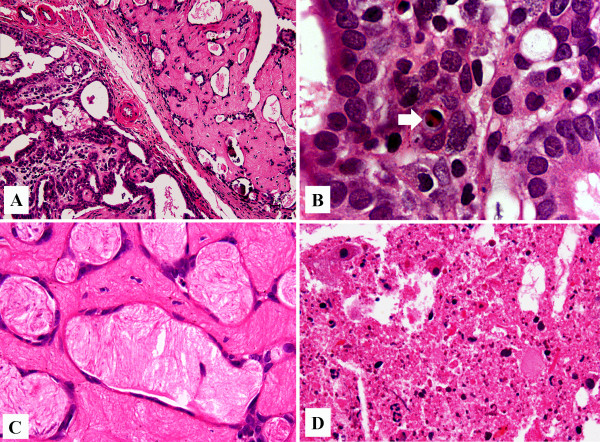
**HE-stained mammary tumor samples at the end of the experimental protocol: (A) mammary tumor border region showing live and degenerative areas, *200x*; (B) live mammary tumor area showing numerous dark basophilic nuclei in epithelial cells surrounding an apoptotic cell (arrow), *1000x*; (C) degenerative mammary tumor area characterized by flattened epithelia and robust stromal hyalinization, *1000x*; (D) necrotic mammary tumor area showing cellular debris, *400x*.**

The histopathological analysis showed that almost all DMBA-induced mammary tumors (>90%) were invasive adenocarcinomas presenting predominantly tubular or papillary patterns (data not shown). The morphometric measurements of live/degenerative/necrotic areas of mammary tumors at the end of the treatment period are shown in Figure 1B and representative images of these histological areas are presented in Figure 2. Mammary tumors from the untreated control group showed a high proportion of live area (80.8%), a moderate proportion of necrotic area (18.7%) and little degenerative area (0.5%) (Figure 1B). The group receiving TAM alone presented a clear change in the morphometric pattern of mammary tumor areas compared to the untreated control group, with 48.8% live, 1.5% necrotic and 36.3% degenerative areas. Co-treatment with 100 mg/kg GbE reduced the proportions of live (24.8%) and necrotic areas (2.9%) (p = 0.046 and p = 0.038, respectively) and significantly increased the proportion of degenerative area (72.9%) (p = 0.004) compared to the group treated only with TAM.

### Immunohistochemical analysis of cell proliferation, apoptosis, ER-α and p63

**Figure 3:**
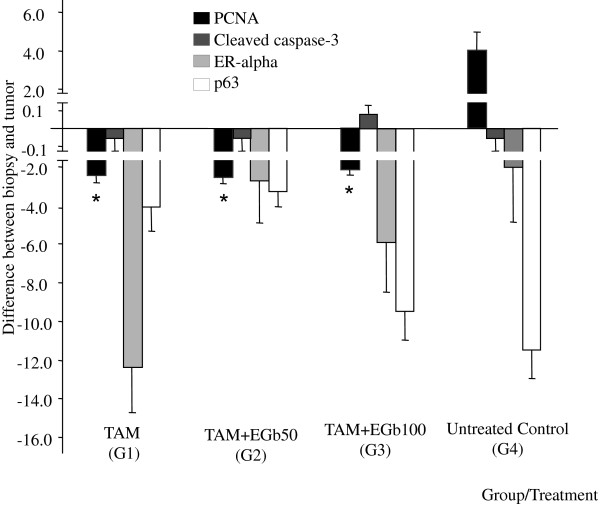
**Immunohistochemical analysis of PCNA (LI%), cleaved caspase-3 (LI%), estrogen receptor-alpha (ER-α, LI%) and p63 (LI%) staining at beginning (referential biopsies) and at the end of the 4-week treatment period (mammary tumors) with tamoxifen and *Gingko biloba *extract. **Data are expressed as the mean ± SEM. TAM = tamoxifen (10 mg/kg, i.p.); GbE 50-100 = *Ginkgo biloba *extract (50 or 100 mg/kg, i.g.). *Significant difference compared to groups G1, G2 and G3, p < 0.005).

**Figure 4:**
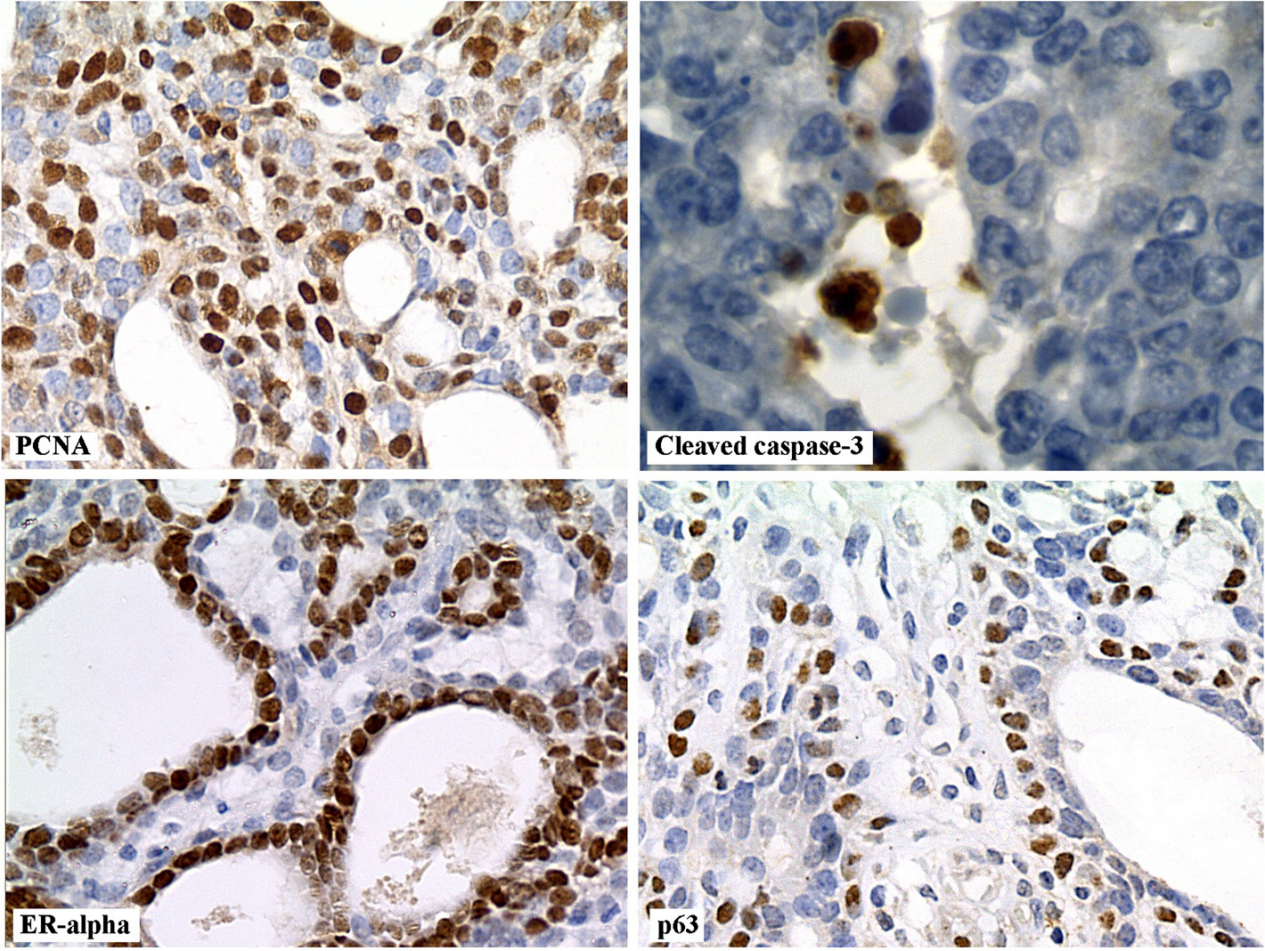
**Representative images from immunohistochemical staining of live tumor areas showing immunoreactivity for PCNA (*400x*), cleaved caspase-3 (*1000x*), ER-α (*400x*) and p63 (*400x*).**

All markers were analyzed within the live areas of mammary tumors at the beginning (referential biopsies) and at the end of treatments period (Figures 3 and 4). The differential expression of PCNA, cleaved caspase-3, ER-α and p63 between referential biopsies at the initial and the tumors at the end of the treatments is shown in Figure 3. A significant reduction in the PCNA labeling index (PCNA LI%) was observed in all TAM-treated groups compared to the respective referential biopsies taken at the beginning of the treatment period. The PCNA LI% within the live tumor areas was significantly higher in mammary tumors from the untreated control group (≥6%) than in those from the TAM-treated groups (≤1%) (p <0.005). Moreover, co-administration of either dose of GbE did not change the anti-proliferative effect of TAM treatment. Similarly, GbE treatment did not affect the expression of ER-α, p63 and cleaved caspase-3 in live tumor tissues compared to the group receiving TAM alone.

## Discussion

The present results indicate that the anti-tumoral properties of Tamoxifen (TAM) were slightly modified by co-treatment with *Ginkgo biloba* extract (GbE) in a DMBA-induced model of mammary carcinogenesis in female Sprague–Dawley rats. Similar to the findings of previous *in vitro*, animal and human cohort studies, we found that oral treatment with TAM induced an anti-tumoral effect characterized by significant mammary tumor regression [6,8,11,23,27,28]. We detected significant reductions in mammary tumor volume, in proportion of living tumor tissue, and in frequency of proliferating cells as indicated by PCNA staining in all TAM-treated groups (G1, G2 and G3). Proliferating cell nuclear antigen (PCNA) is a 36 kDa molecule that acts as a DNA polymerase co-factor during chromosome replication and is easily detected during S-phase of the cell cycle [29]. Therefore, PCNA is widely used as a reliable cell proliferation biomarker in experimental models of chemical carcinogenesis, including models of rat mammary carcinogenesis [24,29,30]. In the present study, the reduced PCNA LI% observed in TAM-treated rats can be explained by the ability of TAM to inhibit estrogen receptor (ER)-dependent cell proliferation in mammary tumors [31]. Co-treatment with 50 or 100 mg/kg GbE did not alter the anti-proliferative effect of TAM in ER-α-positive live tumor tissues, demonstrating that GbE does not compete with TAM for ER binding by acting as an ER agonist or antagonist.

Estrogens play a key role in hormone-sensitive breast cancer cells and their receptors are functional targets of anti-endocrine therapies for breast cancer [32]. Recent cell proliferation assays on MCF-7 cells have demonstrated that extracts of *Ginkgo biloba* have dual effects on endogenous estrogens, as they can act as either agonists or antagonists [33,34]. These pharmacological properties depend on the GbE dose (10–1000 μg/ml) and the concentration of endogenous estrogens present in the cell culture medium [33,34]. The abundant phytoestrogens present in GbE can bind with high affinity to ER, establishing an antagonistic competition with endogenous estrogens such as estradiol (E2) [33,35-37]. Human studies have also shown that both phytoestrogens within GbE and SERMs can antagonize the binding of endogenous estrogens to ERs, increasing the post-surgery survival of the patients [37]. In the present study, we observed a significant change in the histological pattern of mammary tumor areas in rats that received TAM *plus* 100 mg/kg GbE. This group presented a significant increase in the size of degenerative tumor areas together with a reduction in live tumor areas compared to the group receiving only TAM. Although this beneficial effect was observed in rats that received GbE co-treatment, no changes in E2 serum levels were observed, indicating an absence of negative feedback induction. Otherwise, the enhanced tumor regression observed in these rats may be associated with the enhanced bioavailability of TAM when co-administered with quercetin or quercetin-containing dietary supplements such as GbE [38].

The results of the present study showed that treatment with TAM and GbE for 4 weeks induced an outstanding decrease in tumor volume, but complete regression of the tumor was not accomplished. Indeed, islands of ER-α-positive tumor cells remained viable in all TAM-treated groups, suggesting that tumor recurrence would occur upon termination of the treatment. Recent *in vitro*, *in vivo* and human studies have reported *de novo* or acquired resistance to SERMs and AIs [39,40]. TAM itself demonstrates a dual agonist/antagonist activity that results in a weak estrogenic effect, precluding a total blockade of estrogen-stimulated tumor growth [41] and driving the natural selection of TAM-resistant tumor cells [42]. These live areas within mammary tumors display some intrinsic mechanisms that can partially explain their growth in spite of ER signaling, such as increased expression of EGFR and/or HER2 [43]. The cross-talk between ER signaling and growth factor signaling proteins has been proposed to be important for the resistance of tumor cells to endocrine therapies [44]. The overexpression of growth factors and kinase proteins could thus induce an ER-dependent proliferation response in the absence of ER ligands [45].

Caspases are cysteinyl-aspartate specific proteases that belong to the C14 family [46]. The proteolytic cleavage of caspase-2, 3, 6, 7, 8, 9 and 10 in the cytoplasm is directly associated with the regulation and execution of apoptosis [47]. Indeed, cleaved caspase-3 is one of the most commonly used biomarkers for the detection of apoptosis in cell culture and animal and human tissues [48]. In the present study, the cleaved caspase-3 labeling index (cleaved caspase-3 LI%) was not altered by TAM treatment in live tumor areas (i.e., islands of ER-α-positive tumor cells). Therefore, the mode of action of TAM-induced tumor regression in the present study might be mainly related to the ability of TAM to prevent the ER-dependent tumor cells from receiving a proliferation stimulus rather than inducing cell death in mammary tumors. Nevertheless, other *in vivo* studies have demonstrated that different doses of TAM administered for different periods of time display anti-proliferative properties associated with the induction of apoptosis [49]. In addition, co-treatment with 50 mg/kg or 100 mg/kg GbE did not modify apoptosis indexes in mammary tumors treated with TAM, demonstrating that GbE did not affect the anti-proliferative action of TAM. Some *in vivo* studies have detected protective anti-apoptotic effects of *Ginkgo biloba*, but these studies were performed in settings other than cancer [50-53].

p63 is a p53 analogue protein expressed in the nuclei of basal cells such as the myoepithelial cells present in mammary tissue, skin, oral cavity, prostatic and urothelial epithelia [54]. Overexpression of p63 has been frequently observed in squamous cell carcinomas in humans, suggesting that it may function as an oncogene [55]. Indeed, *in vitro* bioassays have demonstrated that gene silencing of some p63 variants modulates the transcription of genes regulated by p53 [54]. Recent studies have demonstrated that the function of myoepithelial cells is strongly associated with the aggressiveness and invasiveness of human breast cancer [56]. Treatment with TAM *plus* GbE did not affect p63 expression in live tumor tissues in the present study.

Various *in vitro* and *in vivo* studies suggest that *Ginkgo biloba* itself has cancer chemopreventive properties, but epidemiological findings are sparse and inconclusive [21,35,57-60]. Thus, the findings of the present study indicate that 4 weeks of treatment with 100 mg/kg GbE had a slightly beneficial effect on the therapeutic efficacy of TAM in female Sprague–Dawley rats bearing mammary tumors.

## Conclusions

Co-administration of GbE during tumor regression in female SD rats receiving TAM was investigated. While TAM induced a robust regression of mammary tumors, GbE had only a slightly additional effect on the anti-tumor efficacy of TAM. Thus, sustained use of GbE by breast cancer patients undergoing treatment with TAM might to safe and/or promote some clinical efficacy.

## Abbreviations

GbE: *Ginkgo biloba* extract; TAM: Tamoxifen; SD: Sprague–Dawley; SERMs: Selective estrogen receptor modulators; AI: Aromatase inhibitors; ER: Estrogen receptor; CAM: Complementary and alternative medicine; COBEA: Brazilian College of Animal Experimentation; HPLC: High performance liquid chromatography; DMBA: 7,12-Dimethyl-benz(a)anthracene; ALT: Alanine aminotransferase; HE: Hematoxylin-eosin; PCNA: Proliferating cell nuclear antigen; LI%: Labeling indexes; DAB: Diaminobenzidine-tetrahydrochroride; E2: Estradiol.

## Competing interests

The authors declare that they have no competing interests.

## Authors’ contributions

LFB and KSF were in charge of the experimental protocol, analysis and interpretation of the results. They participated in the preparation of the manuscript. MAMR was the pathologist of the group. She participated in the analysis, interpretation of the results and in the preparation of the manuscript. MD was responsible for the overall study, from the experimental design to the preparation of the manuscript. All authors read and approved the final manuscript.

## Pre-publication history

The pre-publication history for this paper can be accessed here:

http://www.biomedcentral.com/1472-6882/13/93/prepub
